# Prolonged Gastric Retention of Sodium Zirconium Cyclosilicate: A Case Report

**DOI:** 10.7759/cureus.95106

**Published:** 2025-10-21

**Authors:** Masafumi Yoshinaga, Hiroshi Adachi, Kwonil Choi, Motohiro Shimizu

**Affiliations:** 1 Emergency Medicine, Ryokusenkai Yonemori Hospital, Kagoshima, JPN; 2 Intensive Care, Ryokusenkai Yonemori Hospital, Kagoshima, JPN

**Keywords:** adult gastroenterology, calcium polystyrene sulfonate, sodium polystyrene sulfonate, sodium zirconium cyclosilicate, true hyperkalemia

## Abstract

This is a unique case of prolonged gastric retention of sodium zirconium cyclosilicate (SZC) in a 72-year-old man with multiple comorbidities, including acute kidney injury. Although SZC's radiopacity is a known finding, this patient had no apparent GI motility issues. Serial chest X-rays revealed radiopaque material in his stomach for 11 days after he received the medication. Gastroscopy confirmed that the radiopacity was caused by SZC adhering to the gastric wall, which was successfully removed by irrigation. Following irrigation, imaging showed improved gastric radiopacity. This case highlights that prolonged gastric retention of SZC can occur, potentially increasing the risk of complications. We recommend monitoring the passage of orally administered SZC with imaging studies to prevent such occurrences.

## Introduction

Sodium zirconium cyclosilicate (SZC) is a medication used to treat hyperkalemia. Its unique microporous structure allows it to selectively bind to potassium ions in the GI tract, releasing sodium and hydrogen ions in exchange. This selective binding prevents potassium absorption from the gut, leading to its excretion in the feces and subsequently lowering blood potassium levels. Common side effects of SZC include hypokalemia, edema (swelling), congestive heart failure, and constipation [1-3]. As a drug that acts on the GI tract, SZC may affect GI motility and lead to complications such as stagnation or blockage. Here, we report the first case, to the best of our knowledge, in which SZC remained in the stomach for a long period of time after oral administration, which was confirmed by chest X-ray.

## Case presentation

A 72-year-old man presented with septic shock, acute exacerbation of chronic obstructive pulmonary disease, congestive heart failure, and acute kidney injury. He was intubated upon admission and managed with mechanical ventilation, receiving continuous sedation and analgesia, specifically a continuous intravenous infusion of remifentanil. For stress ulcer prophylaxis, omeprazole was also administered. Enteral nutrition via a gastric tube was started on day two. As blood tests indicated an elevated serum potassium level of 5.5 mmol/L, 30 g/day of Lokelma (SZC) was administered via a gastric tube on days two and three. After SZC administration, his serum potassium level gradually improved to a normal range of 4.8 mmol/L on day four, with no subsequent evidence of hyperkalemia.

**Figure 1:**
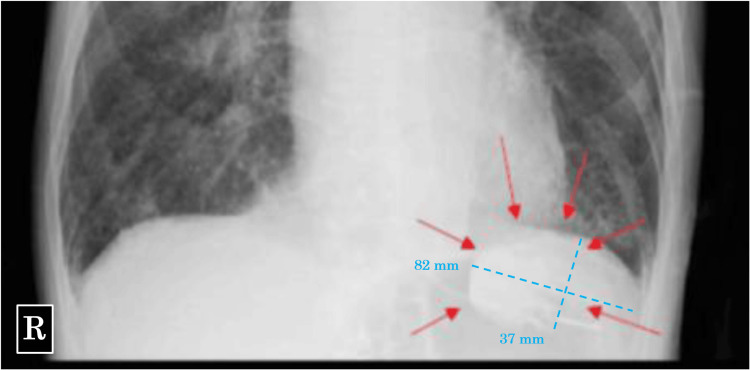
Chest X-ray on day 3 Chest X-ray shows radiopaque material (red arrow) in the stomach.

**Figure 2:**
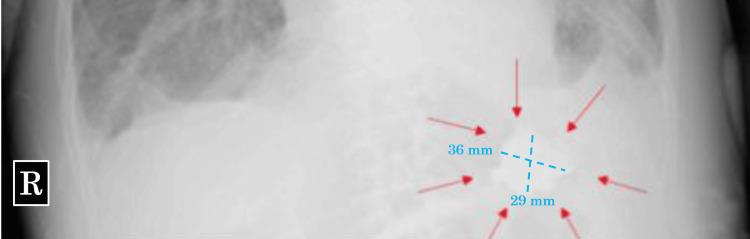
Chest X-ray on day 13 Chest X-ray shows persistent radiopaque material (red arrow) in the stomach.

Daily chest X-rays, performed to monitor for congestive heart failure and respiratory failure, revealed a radiopaque material in the patient’s stomach on day three of admission. Given that SZC was administered on day two and the patient had not received any oral or intravenous radiopaque contrast material, the substance was presumed to be SZC (Figure 1). Although tube feeding proceeded without complications, and the patient continued to have bowel movements with no gastric tube reflux, chest X-rays confirmed the radiopaque substance remained in the same location within the stomach daily until day 13 (Figure 2).

**Figure 3:**
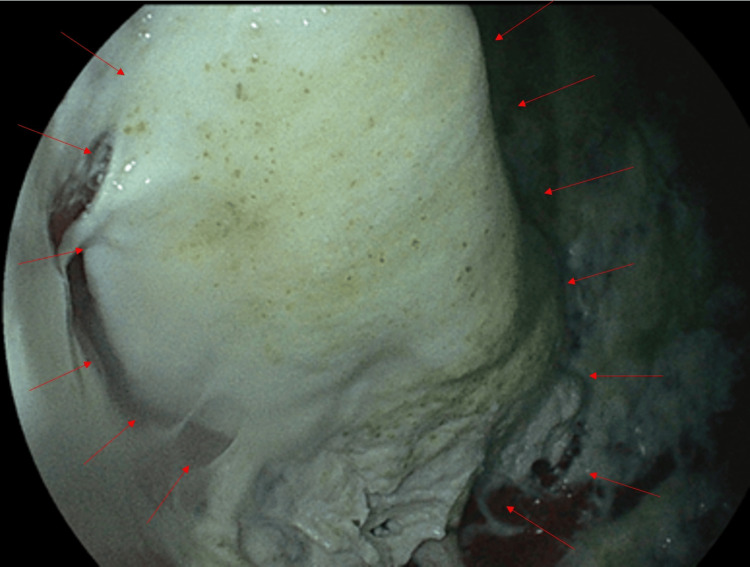
Gastroscopy Gastroscopy shows a thick, pasty suspension, believed to be sodium zirconium cyclosilicate (SZC), adhered to the mucosa of the gastric fundus and upper body (red arrow). No distinct solid mass was observed.

On day 13, gastroscopy confirmed that the substance adhering to the patient’s stomach wall was SZC (Figure 3). We removed the substance by spraying saline under pressure through the endoscope. No radiopaque material was observed in his stomach via chest X-ray post-endoscopy, and no severe GI complications were observed during hospitalization. Despite continued intensive care, the patient died of multiple organ failure on day 19 after admission.

## Discussion

SZC is a novel, non-swelling drug that is reportedly associated with a lower risk of GI complications due to its chemical properties, compared to other potassium binders [4,5]. Other similar drugs like calcium polystyrene sulfonate (CPS) and sodium polystyrene sulfonate (SPS) have been reported to cause intestinal mucositis, constipation, and intestinal perforation [6-8]. In contrast, Holleck et al. reported that SZC, CPS, and SPS were associated with similarly low risks of intestinal ischemia/thrombosis or other serious adverse GI events (Table 1) [9].

**Table 1:** Comparison of GI complications of SZC, SPS, and CPS SZC: sodium zirconium cyclosilicate, SPS: sodium polystyrene sulfonate, CPS: calcium polystyrene sulfonate, GI: gastrointestinal

Feature	SZC	CPS and SPS
Drug type	Novel, inorganic crystal (non-swelling)	Older cation exchange resins
Common GI complications	Constipation (generally mild)	Constipation
Severe GI complications (reported risk)	Generally considered to have a lower risk of serious complications [4,5]	Reported to cause intestinal mucositis and intestinal perforation [6–8]
Mechanism (difference)	Non-swelling chemical properties are hypothesized to contribute to its better safety profile [4,5]	Resins can adhere to the intestinal wall, leading to inflammation and tissue injury
Conflicting data	Holleck et al. reported that SZC, CPS, and SPS were associated with similarly low risks of intestinal ischemia/thrombosis or other serious adverse GI events [9]	Holleck et al. reported that SZC, CPS, and SPS were associated with similarly low risks of intestinal ischemia/thrombosis or other serious adverse GI events [9]

Despite its generally favorable GI safety profile, our case highlights a different concern: gastric retention. The mechanism of the gastric retention and subsequent adhesion in our mechanically ventilated, critically ill patient likely involved a combination of factors.

While SZC's unique properties generally lead to a favorable safety profile compared to older binders, our case highlights a different concern: gastric retention. The mechanism of the gastric retention and subsequent adhesion in our mechanically ventilated, critically ill patient likely involved a combination of factors.

Notably, enteral feeding was tolerated without complications, the patient had bowel movements, and no gastric tube reflux was observed. This suggests the retention was not due to severe ileus or overt gastric paralysis but rather reflects the physical accumulation of the SZC itself. The high water absorption capacity and non-polymeric crystal nature of the drug make it prone to physical clumping and gel formation when mixed with GI fluid.

This risk was likely exacerbated by environmental factors: first, the patient's analgesia regimen included remifentanil, an opioid known to mildly impair GI motility and delay gastric emptying. Second, the co-administration of omeprazole, a proton pump inhibitor (PPI), resulted in an elevated gastric pH. This altered pH environment may have changed the physical properties of the SZC particles, exacerbating their tendency for dense clumping within the stomach.

The combination of mildly impaired motility and the altered pH environment provided the ideal setting for the physical accumulation of the non-absorbed SZC crystals. Once retained, this mass can put pressure on the stomach wall, posing risks such as ulcers or perforation, and may cause symptoms if it obstructs the pylorus. Clinically, this suggests that vigilance is required for upper GI stasis in critically ill patients, even when overt signs of severe ileus, like feeding intolerance or gastric reflux, are absent.

There have been several reports of SZC radiopacity in the GI tract, which is thought to be due to zirconium [10-12]. In our patient's case, since no oral contrast material was administered, we identified the material remaining in the stomach from day three to day 13 on X-ray as SZC. To our knowledge, this represents the first reported case of SZC remaining in the stomach for a prolonged period. We confirmed SZC adhesion to the stomach wall by gastroscopy and had to remove it using water pressure lavage. Shariff et al. reported a rare adverse effect of SPS: a case of acute upper GI bleeding secondary to mucosal ulceration induced by SPS deposition, confirmed by endoscopic evaluation and gastric ulcer histology [13]. Boss et al. reported a case of hemorrhage developing from severe duodenopathy with peptic ulcer disease two days after starting SZC [10]. Prolonged adhesion of SZC to the gastric wall could prolong its contact with the gastric mucosa, potentially leading to the development of acute gastric mucosal lesions and gastric ulcers. Therefore, while our findings confirm the retention itself, a rigorous causality assessment suggests the event was not due to SZC alone but was multifactorial.

The gastric retention in this critically ill patient was likely the result of a confluence of factors: the patient's underlying critical illness, sedation, and mechanical ventilation predisposed them to impaired gastric emptying. This was exacerbated by the co-administered remifentanil, an opioid known to impair motility. Furthermore, the patient received omeprazole, a PPI, creating an elevated gastric pH environment that likely altered the physical state of the SZC particles, promoting dense clumping.

Notably, enteral feeding was tolerated without complications, the patient had bowel movements, and no gastric tube reflux was observed. This suggests the retention was not due to severe ileus or overt gastric paralysis but rather reflects the physical accumulation of the non-polymeric SZC crystals within a high-risk environment. Clinically, this suggests that when orally administering SZC to critically ill patients, it's crucial to acknowledge the compound risk from sedation and PPI use. Furthermore, confirming its passage through the GI tract via daily abdominal X-ray, or utilizing CT abdomen/pelvis as a more sensitive screening method when retention is clinically suspected, is prudent, even when overt signs of severe ileus are absent.

Finally, to assess the causality and severity of this adverse drug reaction, we applied standardized scales. Based on the Naranjo Causality Assessment Scale, the relationship between SZC and gastric retention was rated as "possible" (total score: +4), acknowledging the presence of alternative causes such as critical illness and co-administered medications [14]. Furthermore, the severity of the event, which required intervention (endoscopic removal) and complicated the patient's overall management, was classified as level 4 according to the Hartwig Severity Scale (prolonged length of stay) [15]. These assessments underscore the clinical significance of this unique complication.

## Conclusions

Our case represents, to our knowledge, the first documentation of prolonged gastric retention and adhesion of SZC to the stomach wall, highlighting a novel risk profile distinct from known complications like intestinal perforation. While generally safe, the risk of physical accumulation is heightened in critically ill patients. Clinically, this warrants acknowledging the compound risk associated with the co-administration of medications that impair GI motility, such as opioids, and alter gastric pH, such as PPIs. Consequently, when administering SZC in high-risk settings, it is crucial to confirm its passage through the GI tract via daily abdominal X-ray or by utilizing CT abdomen/pelvis as a more sensitive screening method when retention is clinically suspected. Prompt recognition of this specific type of stasis may necessitate timely endoscopic intervention for removal to prevent further complications.
